# The Expression Profile of Complement Components in Podocytes

**DOI:** 10.3390/ijms17040471

**Published:** 2016-03-30

**Authors:** Xuejuan Li, Fangrui Ding, Xiaoyan Zhang, Baihong Li, Jie Ding

**Affiliations:** Department of Pediatrics, Peking University First Hospital, Beijing 100034, China; clairesnow@126.com (X.L.); youngbear@126.com (F.D.); wserien@163.com (X.Z.); baihongli89@126.com (B.L.)

**Keywords:** podocyte, complement expression, podocyte injury

## Abstract

Podocytes are critical for maintaining the glomerular filtration barrier and are injured in many renal diseases, especially proteinuric kidney diseases. Recently, reports suggested that podocytes are among the renal cells that synthesize complement components that mediate glomerular diseases. Nevertheless, the profile and extent of complement component expression in podocytes remain unclear. This study examined the expression profile of complement in podocytes under physiological conditions and in abnormal podocytes induced by multiple stimuli. In total, 23/32 complement component components were detected in podocyte by conventional RT-PCR. Both primary cultured podocytes and immortalized podocytes expressed the complement factors C1q, C1r, C2, C3, C7, MASP, CFI, DAF, CD59, C4bp, CD46, Protein S, CR2, C1qR, C3aR, C5aR, and Crry (17/32), whereas C4, CFB, CFD, C5, C6, C8, C9, MBL1, and MBL2 (9/32) complement factors were not expressed. C3, Crry, and C1q-binding protein were detected by tandem mass spectrometry. Podocyte complement gene expression was affected by several factors (puromycin aminonucleoside (PAN), angiotensin II (Ang II), interleukin-6 (IL-6), and transforming growth factor-β (TGF-β)). Representative complement components were detected using fluorescence confocal microscopy. In conclusion, primary podocytes express various complement components at the mRNA and protein levels. The complement gene expressions were affected by several podocyte injury factors.

## 1. Introduction

Complement components comprise approximately 40 serum proteins, glycoproteins that exhibit enzymatic activity, and soluble or membrane-bound receptors; these proteins have long been appreciated as major effectors of the innate immune response [1]. Most complement components are produced in the liver [2]. In recent years, many studies have shown that extrahepatic tissues, including the kidney, brain, blood vessels, lungs, intestines, joints, and skin, can synthesize small amounts of complement components [3,4]. Among extrahepatic tissues, the kidney is one of the main sites of complement synthesis [2,4].

Evidence increasingly indicates that the complement system plays a pivotal role in mediating renal diseases. Several studies have demonstrated the effect of circulating complements on causing primary and secondary renal diseases, including membrane proliferative glomerulonephritis, IgA nephropathy, lupus nephritis, and atypical hemolytic uremic syndrome [5,6,7,8,9]. Abbate *et al.* [10] suggested that ultrafiltered C3 contributes more to tubulointerstitial damage than locally-synthesized C3 in a model of proteinuric progressive nephropathy. However, recent evidence also suggested that locally-expressed complement proteins are involved in kidney tissue injury [11]. Tang *et al.* found that complement proteins are synthesized in the kidney, thus contributing significantly to the circulating pool of C3 [12], the central protein of the complement cascade. Other studies reported increased C3 expression during renal inflammation [13] and in proteinuric diseases [14]. In some kidney diseases, histological examination demonstrated a spatial relationship between tissue injury and complement protein deposition [15,16,17]. Furthermore, in studies of a proteinuric nephropathy model, complement deficiency or complement inhibition were found to reduce the degree of histological injury and to reduce the loss of renal function. Recently, Sheerin *et al.* [18] analyzed the expression of complement components in a model of adriamycin-induced proteinuria to determine the effect of locally-synthesized C3. They found that kidney isografts from C3 knock-out mice, when transplanted in wild-type mice, were protected from proteinuria-associated complement activation, tubular damage, and progressive renal failure, despite the presence of abundant circulating C3, because adriamycin nephropathy is characterized by glomerular podocyte injury, including foot process effacement and podocyte loss [19]. In addition, significantly staining C3 was demonstrated in glomeruli from mice with adriamycin nephropathy when compared saline-injected control mice. All of these indirectly indicate that lack C3 in renal podocytes reduces early glomerular injury and proteinuria and ameliorates subsequent glomerular and tubulointerstitial scarring with the preservation of renal function. Therefore, we consider that the complement production in podocytes is important for the development proteinuric glomerulopathies. Nevertheless, no direct evidence supports the suggestion that podocytes express complement proteins.

Podocytes are critical for maintaining the glomerular filtration barrier and are the target cell of injury in proteinuric renal diseases, such as minimal change nephrotic syndrome (MCNS), focal segmental glomerulosclerosis (FSGS), and membranous nephropathy (MN) [20]. The complement proteins that are expressed in podocytes and changes in complement expression that occur during podocyte injury are not known. Interestingly, in our previous study, we found that the expression of some complement components was significantly up-regulated in a rat nephropathy model at times corresponding to the effacement of podocyte foot processes and the development of proteinuria [21]. In addition, several studies have indicated that podocytes can express complement components such as CR1 (complement receptor 1) [22,23], C3 [24], C4 [25], CFH (complement factor H) [26], and DAF (decay accelerating factor) [27]. However, the profile and extent of complement component expression in podocytes remain unknown. Thus, this study aimed to obtain direct evidence of complement expression by primary cultured podocytes and to determine the profile of complement components that are expressed in podocytes under physiological conditions and during podocyte injury induced by various stimuli.

## 2. Results

### 2.1. Complement Gene Expression in Podocytes

**Figure 1:**
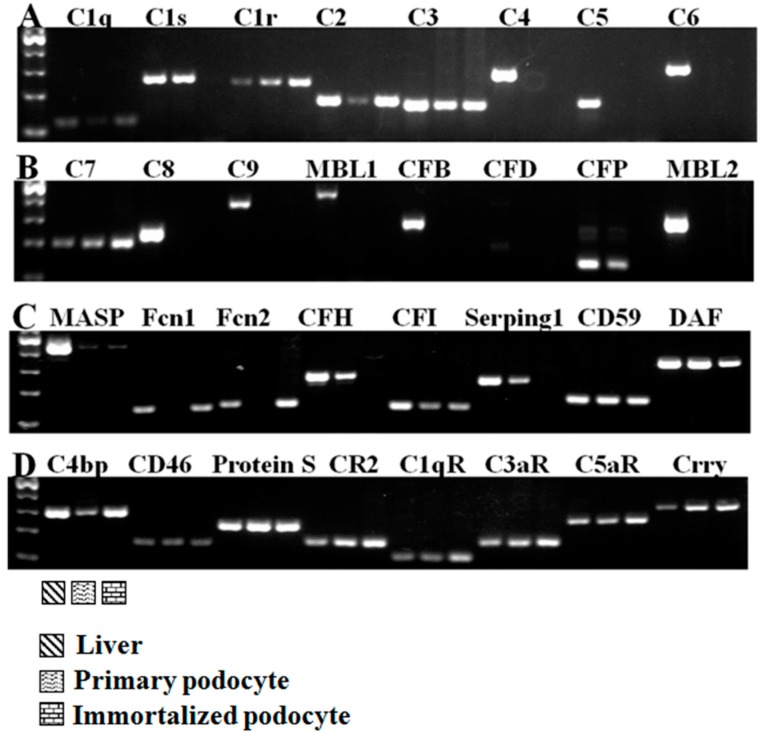
The expression of complement genes in primary cultured murine podocytes and immortalized murine podocytes. (**A**–**D**) Primary cultured podocytes and immortalized murine podocytes expressed the complement factors C1q, C1r, C2, C3, C7, MASP, CFI, DAF, CD59, C4bp, CD46, Protein S, CR2, C1qR, C3aR, C5aR, and Crry. Neither the primary nor the immortalized podocytes exhibited specific bands for C4, CFB, CFD, MBL1, MBL2, C5, C6, C8, or C9 (9/32). Total RNA from mouse liver tissue was used as a positive control. *n* = 3.

We examined the expression of 32 complement components, including inherent complement components, complement regulatory factors, and complement receptors (Figure 1). Under normal culture conditions, primary cultured podocytes expressed 21/32 complement genes, and immortalized murine podocytes expressed 19/32 complement genes. As shown in Figure 1, primary cultured podocytes and immortalized murine podocytes all expressed the complement factors C1q, C1r, C2, C3, C7, MASP, CFI, DAF, CD59, C4bp, CD46, Protein S, CR2, C1qR, C3aR, C5aR, and Crry (17/32). Neither the primary nor the immortalized podocytes exhibited specific bands for C4, CFB, CFD, MBL1, MBL2, C5, C6, C8, or C9 (9/32) (Figure 1A,B). However, the expression of some complement components was inconsistent. The primary cultured podocytes expressed complement C1s, CFP, CFH, and Serping 1, whereas the immortalized murine podocytes expressed complement Fcn1 and Fcn2. Therefore, podocytes express many complement factor genes.

### 2.2. Complement Protein Expression in Podocytes Determined Using Liquid Chromatography–Mass Spectrometry/Mass Spectrometry (LC–MS/MS) Analysis

Proteins perform various biological functions in the cell. To further identify complement protein expression in podocytes, we used tandem mass spectrometry to determine the normal podocyte profile. We identified 3296 proteins (see Table S1). The number of complement-related gene proteins is shown in Table 1.

### 2.3. The Effect of Multiple Stimulating Factors on Complement Gene Expression

**Figure 2:**
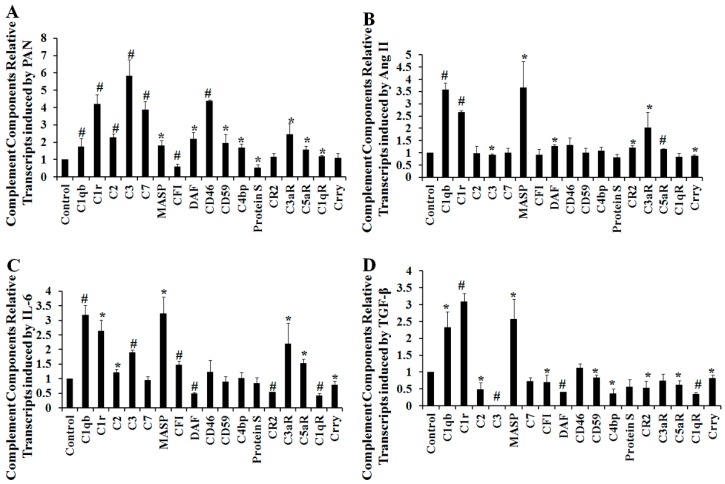
The effect of stimulation by various factors on complement gene expression. Cultured immortalized podocytes were treated with 50 μg/mL puromycin aminonucleoside (PAN) (**A**); 10^−6^ M angiotensin II (Ang II) (**B**); 100 ng/mL interleukin-6 (IL-6); (**C**) or 5 ng/mL transforming growth factor-β (TGF-β) (**D**). Complement gene expression was quantitatively analyzed using quantitative real-time RT–PCR or conventional RT-PCR. Data are presented as means ± SD. *n* = 3. * *p* < 0.05 *vs.* control group; ^#^ *p* < 0.01 *vs.* control group.

Having shown that podocytes express many complement genes under normal physiological conditions, we sought to understand how the expression of these genes was affected after stimulation with podocyte injury factors. Cultured podocytes were treated with 50 μg/mL puromycin aminonucleoside (PAN), 10^−6^ M angiotensin II (Ang II), 100 ng/mL interleukin-6 (IL-6), or 5 ng/mL transforming growth factor-β (TGF-β). Complement gene expression was quantitatively analyzed using quantitative real-time RT-PCR. Several complement genes were regulated after podocyte injury (Figure 2A–D), but not C4, CFB, CFD, MBL1, MBL2, C5, C6, C8, nor C9.

### 2.4. The Effect of Puromycin Aminonucleoside (PAN) on Complement Protein Expression as Determined Using iTRAQ LC–MS/MS Analysis

**Figure 3:**
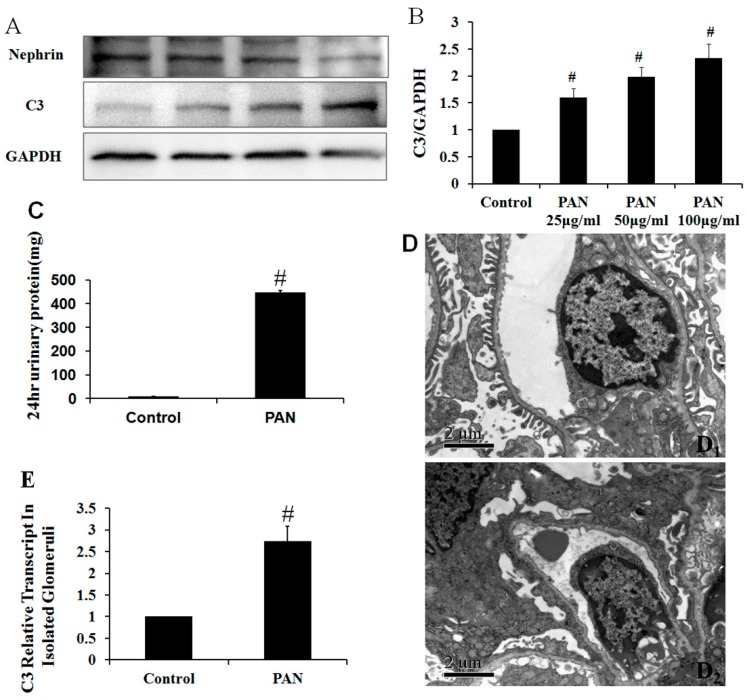
C3 expression increased in podocytes after PAN treatment for 24 h and in isolated glomeruli. (**A**) The C3 protein level was determined by Western blot analysis; (**B**) The protein amounts were quantified and normalized to GAPDH expression. *n* = 3; (**C**) Compared to the control groups, twenty-four hour urine protein levels increased significantly at day 10 after PAN injection; (**D_1_**) Ultrastructural analysis showed that the foot processes were long and thin in control group rats; (**D_2_**) Ten days after PAN injection, the foot processes were no longer present, and diffuse and widespread fusion was observed. Scale bar: 2 mm; (**E**) C3 mRNA expression levels were evaluated by quantitative real-time PCR in isolated glomeruli. Data are presented as means ± SD. *n* = 5. ^#^ *p* < 0.01 *vs.* control group.

The expression of complement genes in podocytes was regulated under several podocyte injury conditions. The use of isobaric tags for relative and absolute quantification (iTRAQ) in combination with multidimensional liquid chromatography (LC) and tandem MS analysis is a powerful tool for quantitative proteomic analysis. Differences in protein expression after treatment with 50 μg/mL PAN was quantified using an iTRAQ-based proteomics approach. To assess variation in the iTRAQ quantification experiment, we compared the ratio of differentially-expressed proteins in the treatment group to that in the control group (see Table S2). Among differentially-expressed proteins, only complement C3 was expressed at higher levels after PAN-induced podocyte injury (Table 2). To confirm the iTRAQ result, Western blot was used to determine the complement C3 level after PAN dose-dependent induced podocyte injury. The C3 protein levels were significantly higher after injury (Figure 3A,B). In addition, we also constructed PAN-induced rat nephropathy model. Proteinuria levels and foot processes in the PAN rat nephropathy model were shown in Figure 3C,D. C3 mRNA expression levels were evaluated by quantitative real-time PCR in isolated glomeruli. The C3 protein levels were also significantly higher after injury in Figure 3E.

### 2.5. Multiple Complement Factor Expression and Distribution in PAN-Induced Rat Nephropathy

**Figure 4:**
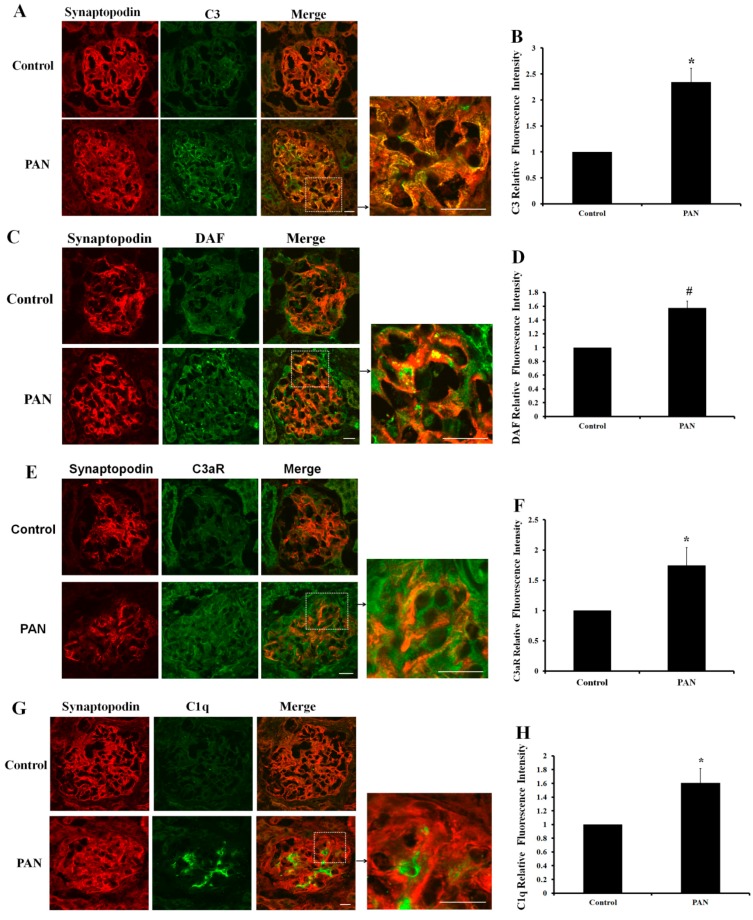

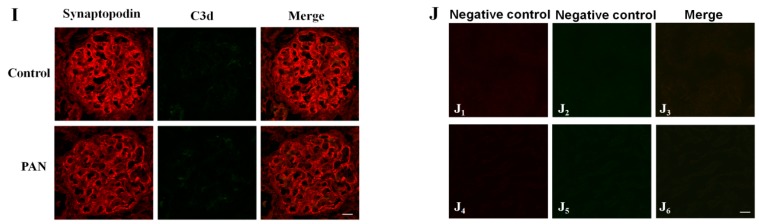
Representative complement immunofluorescent staining in the PAN rat nephropathy model. Immunofluorescent staining of C3 (**A**), DAF (**C**), C3aR (**E**), C1q (**G**), and C3d (**I**), with synaptopodin in a PAN nephropathy rat model. Complement factors are labeled in green, and synaptopodin is labeled in red. Scale bar for overview: 20 μm; higher magnification: 30 μm. (**B**,**D**,**F**,**H**) Immunofluorescence staining of C3, C3aR, DAF, and C1q in glomeruli, the positive signals were quantified as the relative fluorescence intensity. Each column represents the mean value that was derived from four glomeruli in a single experiment. The results are presented as the mean value of three independent experiments; (**J**) Images **J**_**1**–**6**_ are the negative controls by without primary antibodies; images **J_1_**_,**2**,**4**,**5**_ were incubated with Alexa Fluor^®596^ goat anti-mouse IgG, Alexa Fluor^®488^ goat anti-rabbit IgG, Alexa Fluor^®596^ goat anti-rabbit IgG, and Alexa Fluor^®488^ goat anti-mouse IgG, respectively. Data are presented as means ± SD. * *p* < 0.05 *vs.* control group; ^#^ *p* < 0.01 *vs.* control group.

Proteomics is a relatively novel technology, but iTRAQ is insufficiently sensitive to detect proteins that are expressed at lower abundance. However, the results indicated that several complement factors might be involved in podocyte injury. Therefore, we chose five representative complement factors with consistent expression profiles in primary and immortalized murine podocytes for further study. Renal cortex cryosections from the PAN rat nephropathy model were used to assess complement immunofluorescence. Immunofluorescent staining showed that C3 expression increased significantly by day 10 after PAN injection compared with the control (Figure 4A,B). Synaptopodin, an actin-binding protein, is a podocyte-specific marker. Double-labeling assays showed that C3 has co-localized well with synaptopodin (Figure 4A). Complement receptor and complement regulation factors also play important biological functions in the complement system. Although DAF and C3aR were mainly detected in endothelial cells and mesangial cells, the co-localization of complement (DAF and C3aR) with synaptopodin exists and can also be observed (Figure 4C,E). After PAN treatment, DAF and C3aR expression increased (Figure 4D,F). C1q expression was very weak in podocytes. By day 10 after PAN injection, the expression of this protein was significantly higher, but it did not colocalize with synaptopodin and was mainly found in mesangium (Figure 4G,H). The expression and distribution of the activated C3 cleavage fragment C3d did not change, as shown in Figure 4I. It suggests that C3 was not activated in the PAN rat nephropathy. These observations indicate that complement factors, especially complement C3 and C3aR, are involved in PAN-induced podocyte injury.

## 3. Discussion

Increasing evidence has demonstrated the ability of native kidney cells, including endothelial, epithelial, and tubule cells, to produce many complement proteins [28,29,30]. This study showed that murine primary cultured podocytes express several complement genes, including inherent complement components, complement regulators, and complement receptors. Although the podocytes producing the complement components were not all of complement components, there were studies also reported that some extra hepatocyte cells did not produce all of complement components. Such as the primary retinal pigment epithelium cells, Luo *et al.* found that retinal pigment epithelium can express complement genes C1r, C1s, C2, C3, C4, MASP1, CFB, and CFH, but not express C1qb, MBL1, MBL2, C5, or CFI [31]. Moreover, we also measured protein expression under normal physiological conditions using LC–MS analysis. Complement mRNA analysis by PCR, which has high sensitivity and good specificity. Thus, we obtained broad profiles of complement components at the mRNA level. The LC–MS analysis only detected complement C3, Complement component receptor 1-like, and complement component 1 Q subcomponent-binding protein expression in podocytes. Although the LC–MS-based proteomics is a powerful technique for the profiling of protein expression in cells in a high-throughput fashion [32,33], it is challenging to detect lower-abundance proteins in complex mixtures for LC–MS [34,35]. Therefore, in our study, we detected complement proteins with higher abundance and the most significant changes of complements after podocyte injury. In addition, the expression of mRNA and protein is not strictly consistent. Different regulation mechanisms (such as synthesis and degradation rates) could be involved in the mRNA transcription and protein translation, which results in different expression profiles, as well as expression levels of mRNA and proteins [36]. In addition, several studies have indicated that podocytes express some complement components, such as CR1 [22,23], C3 [24], C4 [25], CFH [26], and DAF [27]. C4 was not detected in primary podocytes. The discrepancy between C4 gene expression in primary cultured podocytes in humans and mice might reflect a species difference. Our study demonstrated that podocytes synthesize complement components, and we further measured the podocyte complement expression profile.

The major classic complement pathway genes C1qb, C1r, C1s, and C2 were expressed. Regarding the alternative pathway and the mannan binding lectin (MBL) pathway, only low levels of mannan-binding lectin-associated serine protease (MASP) and complement factor P (CFP) expression were detected in primary podocytes. Complement C3 and C5 are essential for the full activation of the complement system in all three pathways. Interestingly, C3, but not C5, was expressed in the primary podocytes. Our results suggest that some components that are crucial for the formation of the membrane attack complex (MAC) are missing, including C4, C5, C6, C8, and C9 (Figure 1). The lack of these indispensable components directly led to the incapability of forming MAC. Therefore, there was no downstream biological function through the classical complement cascade.

Quantitative real-time PCR analysis demonstrated that the production of complement proteins is under the control of several podocyte injury factors, including PAN, Ang II, IL-6, and TGF-β, suggesting that local factors within the kidney might control local levels of complement protein expression. This notion is supported by the observation that intrarenal complement gene expression is increased during inflammatory renal injury in both native and transplant kidney diseases [37,38]. Animal studies have provided confirmatory evidence that local complement production increases during disease development, and a temporal association has been found between the development of injury and increased complement gene expression [39]. We also measured protein levels after stimulation with several podocyte injury factors. An iTRAQ analysis only showed an increase in C3 expression after PAN treatment. Further confirming the iTRAQ results, Western blotting demonstrated that C3 protein was significantly, and dose-dependently, increased after PAN stimulation of podocytes.

No reports have yet described how the expression of complement proteins in podocytes might contribute to the pathogenesis of PAN-induced nephropathy. The use of complement gene knockout mice has indicated that complement molecules might play a protective or injurious role in the pathogenesis of other proteinuria models [40,41,42]. The results of our study demonstrated that primary cultured podocytes and immortalized murine podocytes express the complement factors C1q, C1r, C2, C3, C7, MASP, CFI, DAF, CD59, C4bp, CD46, Protein S, CR2, C1qR, C3aR, C5aR, and Crry. Although the expression of complement profile was obtained from the primary cultured podocyte, there are some limitations about the primary podocytes which were obtained from the terminator mouse model by diphtheria toxin selection. PAN exposure increased C3, DAF, and C3aR expression. These proteins are inherent complement components, complement regulators, and complement receptors. Although the immunofluorescence staining results suggested that C3 was significantly increased in the PAN nephropathy model, it did not completely rule out the sources of C3 from serum. However, the quantitative real-time PCR and Western blot results demonstrate that the C3 mRNA and protein level was significantly increased in podocyte cell-line after PAN treatment. The results further demonstrated that the increased complement C3 was produced by the injured podocyte. In the present study, complement factor functions have not been reported for renal function. However, emerging evidence suggests that intragraft local complement activation contributes to progressive kidney injury [18].

C3 is implicated in the activation of the renin-angiotensin system and of the epithelial-to-mesenchymal transition [43,44]. Furthermore, Sheerin *et al.* [18] found that kidney isografts from C3 knock-out mice, when transplanted in wild-type mice, protected from proteinuria-associated complement activation, tubular damage, and progressive renal failure, despite the presence of abundant circulating C3. Adriamycin nephropathy is characterized by glomerular podocyte injury. Podocytes might synthesize complement components that mediate glomerular diseases. The results of our study directly demonstrate that complement might be produced by podocytes to mediate renal injury. Only one study investigated complement regulators. The researchers found that podocytes express CFH/complment factor H related proteins (CFHRs) at different levels. CFH binding to podocytes mediates cellular co-factor activity and thereby has the potential to locally de-regulate complement activity [45]. Elucidating the local role and synthesis of these proteins might help in understanding their pathophysiological role in renal diseases. Reports describing complement receptors mainly focus on C3aR and C5aR, and recent studies suggest that locally-generated complement components and their active products, such as C3a and C5a, might contribute to pathological processes in inflammatory and immunological diseases. In our study, we measured C3aR and C5aR expression in primary cultured podocytes. Thus far, no studies have reported the involvement of C3aR and C5aR in podocyte injury regulation. *In vivo*, C3a secreted by podocytes might have direct effects on tubular epithelial cells and collagen gene expression [44]. Moreover, some studies have reported that the absence/blockade of C5/C5aR (but not the blockade of MAC formation) limits kidney fibrosis in several animal models [46,47], suggesting that kidney-derived complement C5aR participates in fibrosis in native and transplanted kidneys. In addition, in our study, the expression of C1q has a discrepancy between in cultured podocytes and in the PAN model. It might come from the restricted control condition in the experiment *in vitro*. Even so, the expression of C1q was barely increased in cultured podocytes. However, the local function of complements in kidney cells, especially podocytes, warrants further study.

Therefore, the podocyte could express multiple complement components. We directly demonstrated complement C3 was increased after podocyte injury by PAN *in vivo* and *in vitro*, which indicated that the increased C3 is not just an accompanying phenomenon and could contribute directly to the pathogenesis of the PAN nephropathy. However, the significance of increased C3 in glomerular podocytes and the underlying molecular pathomechanism in proteinuric kidney disease are important questions that remain unclear. In order to explore the role of podocyte-derived C3 involving podocyte injury, as well as in proteinuric kidney diseases, further investigation is needed, such as generating a podocyte-specific C3 (Podo-C3) transgenic mouse.

## 4. Materials and Methods

### 4.1. Animals

The animal studies were approved by the Animal Research Review Board of Peking University (Application No.: 201226; Decission No.: J201226; Date: 10 August 2012). The terminator (*Podocin-Cre;Rosa-DTR^flox^*) mice were kindly provided by Lloyd G. Cantley (Yale University School of Medicine, New Haven, CT, USA). Male Sprague–Dawley rats (120–140 g) were purchased from the Experimental Animal Center at Peking University Health Science Center and divided into two groups. The first group (*n* = 5) was treated with normal saline, and the second group (*n* = 5) received a single intraperitoneal injection of puromycin aminonucleoside (PAN) (15 mg/100 g body weight, Sigma, St. Louis, MO, USA), as previously described [20]. All animals were killed at day 10 after PAN treatment.

Twenty-four hour urine was collected at day 10, and urinary protein was measured using an automatic biochemical analyzer (7170A, Hitachi, Tokyo, Japan) and a pyrogallol red-molybdate dye-binding method. All rats were killed on day 10, and the kidneys were removed. The renal cortex of one kidney was divided into four parts: one part was fixed in 3% glutaraldehyde for examination under transmission electron microscopy, a second part was embedded in Optimum Cutting Temperature (OCT) compound (Sakura, Tokyo, Japan) for examination under fluorescence confocal microscopy, and the remaining two parts were stored at −80 °C. The other kidney was used to isolate glomeruli using the differential sieving method [21].

Transmission electron microscopy was used to evaluate the ultrastructural changes of glomerular podocytes. The renal cortex that was stored in 3% glutaraldehyde was further fixed in 1% osmium tetroxide and then dehydrated in graded ethanol, washed in acetone, and embedded in Epon 812. Ultrathin sections were stained with uranyl acetate and lead citrate and examined under a transmission electron microscope (JEM-1230, JEOL, Tokyo, Japan).

### 4.2. Cell Culture

High-purity primary podocyte cultures were obtained from the terminator (*Podocin-Cre; Rosa-DTR^flox^*) mice using a previously described method with slight modifications [48]. Briefly, mouse kidneys were cut into small pieces and incubated with 1 mg/mL type I collagenase (Sigma-Aldrich, Dorset, UK) at 37 °C for 45 min with occasional agitation; the cell suspension was then filtered through a 40-μm cell strainer (BD Biosciences, Oxford, UK) and seeded onto multiple rat tail collagen pre-coated plates. Forty-eight hours after seeding, medium containing diphtheria toxin (Sigma, St. Louis, MO, USA, 100 ng/mL) was applied to the culture for two weeks.

Immortalized mouse podocytes (MPC5, a generous gift from Peter Mundel, Boston, MA, USA) were cultured under growth-permissive conditions on rat tail collagen type I-coated plastic dishes (Corning, Franklin Lakes, NJ, USA) at 33 °C in RPMI 1640 medium (Invitrogen, Carlsbad, CA, USA) supplemented with 10% fetal bovine serum (Gibco BRL, Gaithersburg, MD, USA), 10 U/mL mouse recombinant γ-interferon (Sigma, St. Louis, MO, USA), 100 U/mL penicillin and 0.1 mg/mL streptomycin (Gibco BRL, Gaithersburg, MD, USA). Podocytes were grown in a flask and incubated at 37 °C under 5% CO_2_ for a minimum of 10–14 days to allow the cells to differentiate.

### 4.3. RNA Isolation and Reverse Transcription

Total RNA was extracted from tissues or cultured cells using TRIzol reagent (Invitrogen, Carlsbad, CA, USA). An RNase-free DNase kit (Qiagen Ltd., Dusseldorf, Germany) was used for the optional DNase treatment. The quantity and quality of the RNA was determined using a NanoDrop ND-1000 spectrophotometer (NanoDrop Technologies, Wilmington, DE, USA). First-strand cDNA synthesis was performed by reacting 2 μg of total RNA with a random primer using the Super Script™ II Reverse Transcriptase kit (Invitrogen, Paisley, UK).

### 4.4. Conventional Reverse Transcription Polymerase Chain Reaction (RT-PCR)

Conventional RT–PCR was performed in primary cultured podocytes and immortalized murine podocytes to measure complement component mRNA expression levels. The primers were designed using NCBI Primer-BLAST and are listed in Table 3. The PCR products were resolved using agarose gel electrophoresis. Several complement components in mouse liver tissue were amplified as a positive control.

### 4.5. Treatment of Podocytes with Various Factors

The effect of puromycin aminonucleoside (PAN, 50 μg/mL), angiotensin II (Ang II, 10^−6^ M), interleukin-6 (IL-6, 100 ng/mL), or transforming growth factor-β (TGF-β, 5 ng/mL) on the expression of complement genes and proteins was measured *in vitro* in the mouse podocyte line using quantitative real-time PCR. The cells were also subjected to total RNA and total protein extraction. Triplicate samples were measured in each treatment. The concentrations used in this experiment were based on results reported in previous podocyte injury studies by us and others [49,50,51].

### 4.6. Quantitative Real-Time PCR Analysis

Quantitative real-time PCR was performed using SYBR Green (Applied Biosystems, Carlsbad, CA, USA). The primers used to amplify complement genes are shown in Table 1. PCR reactions were performed on a Bio-Rad (Bio-Rad, Hercules, CA, USA) thermal cycler. The expression level of each of the complement genes was normalized to GAPDH in each specimen.

### 4.7. Protein Extraction, Digestion, and Labeling with iTRAQ Reagents, LC–MS/MS, and Database Searches

Total protein contents in the podocyte injury samples were 0.64, 0.58, 0.70, 0.68, and 0.79 mg/mL for the control and the samples treated with 50 μg/mL PAN, 10^−6^ M Ang II, 100 ng/mL IL-6, and 5 ng/mL TGF-β, respectively. The mixtures were centrifuged at 15,000× *g* for 15 min at 4 °C. The supernatant was collected, and the protein concentration was determined using the Bradford protein assay (Bio-Rad Laboratories, Hercules, CA, USA). Then, 100 μg of protein was mixed overnight with four volumes of cold (−20 °C) acetone and then dissolved using dissolution buffer. After reduction, alkylation, and trypsin digestion, the samples were labeled following the manufacturer’s instructions as described in the iTRAQ protocol. The labeled samples were pooled for further analysis.

The iTRAQ-labeled sample mixtures were then fractionated using strong cation exchange (SCX) chromatography using a chromatographic column (Phenomenex Luna SCX 100A, 250 × 4.6 mm internal diameter (i.d.), filler particle diameter: 5 μm; Torrance, CA, USA) mounted on a high-performance liquid chromatography (HPLC) system (3220; RIGOL, Beijing, China). Mobile phase A consisted of 25% ACN and 10 mM KH_2_PO_4_ (pH 3.0). Mobile phase B consisted of: 25% ACN, 2 M KCL, and 10 mM KH_2_PO_4_ (pH 3.0). The solvent gradient was as follows: 0%–5% B for 1 min, 5%–30% B for 20 min, 30%–50% B for 5 min, and 50%–100% B for 5 min. The output was measured at 214 nm. Peptides were collected every minute within the effective gradient from 5% to 30%. Thirty-six fractions were collected and desalted using a Strata-X C18 column (Phenomenex, Torrance, CA, USA). The dried fractions were dissolved in 0.1% formic acid (FA) aqueous solution and combined into 16 samples. The samples were centrifuged, and the supernatant was collected. The supernatant was then analyzed using the Dionex-U3000 (Thermo Fisher, Schuylerville, NY, USA) liquid phase system interfaced with a Q Exactive mass spectrometer (Thermo Fisher, Schuylerville, NY, USA).

Data were acquired for 38 min. The spray voltage used was 2.0 KV, and the acquisition quality range was 350–2000 Da.

Protein identification and relative quantification were performed using Protein Discoverer software (version 1.3, Matrix Science, London, UK).

### 4.8. Western Blot

The cells samples were lysed on ice in RIPA lysis buffer containing 50 mM Tris–HCl pH 7.4, 150 mM NaCl, 1% Triton X-100, 1% sodium deoxycholate, 0.1% SDS and complete protease inhibitor cocktail (sodium orthovanadate, sodium fluoride, EDTA, and leupeptin) for 10 min. Cell lysates were collected and quantified using a BCA protein assay kit. Equal amounts (50 μg) of protein from each sample were resolved on 5%–10% SDS-PAGE gels and transferred to nitrocellulose membranes for 1.5 h. After a final washing step, the membranes were developed using enhanced chemiluminescence reagent (Millipore, Bedford, MA, USA). The total gray value of each band was digitized using ImageJ2x software (NIH, Bethesda, MD, USA). The relative expression level of each protein was normalized to that of GAPDH, and the resulting ratios for the control group were normalized to 1.

### 4.9. Fluorescence Confocal Microscopy

Five-micrometer cryosections were fixed in ice-cold acetone, subsequently permeabilized and blocked with 0.3% Triton X-100 and 10% goat serum. The following primary antibodies were used: rabbit anti-C3, DAF, C3aR (1:25, Santa Cruz, CA, USA), mouse anti-C1q (1:50, Abcam, Cambridge, MA, USA), mouse anti-C3d (1:50, Abrova, Taipei, Taiwan), mouse anti-synaptopodin (1:100, PROGEN, Heidelberg, Germany), rabbit anti-synaptopodin (1:100, Abcam, Cambridge, MA, USA), and rabbit anti-nephrin (1:1000, Sigma, St. Louis, MO, USA). After washing three times, the slides were incubated with Alexa Fluor^®488^goat anti-rabbit IgG, Alexa Fluor^®596^goat anti-rabbit IgG, Alexa Fluor^®488^ goat anti-mouse IgG and Alexa Fluor^®596^ goat anti-mouse IgG (1:200, Invitrogen, Carlsbad, CA, USA). The slides were mounted using 15% Mowiol (Sigma, St. Louis, MO, USA). Stained images for each antibody were obtained at the same light exposure using confocal laser-scanning microscopy (Zeiss Lsm510 Meta, Jena, Germany). Images of podocytes stained with each antibody were selected randomly and analyzed by a person who was blind to the study groups.

### 4.10. Statistical Analysis

All data are expressed as means ± SD. ANOVA was used to compare differences between multiple groups. Differences were considered statistically significant at *p* < 0.05.

## Figures and Tables

**Table 1:** List of complement component proteins identified using LC–MS/MS analysis.

Accession Number Protein Name Protein Score	Protein Molecular Weight (*M*_W_)	Protein Isoelectric Point (PI)
P01027 complement C3 43.17	186.4	6.73
Q64735 complement component 36.46 receptor 1-like protein	53.7	6.65
O35658 complement component 1 Q 171.45 subcomponent-binding protein	31	4.92

**Table 2:** List of significantly differentially-expressed proteins identified by iTRAQ analysis of PAN-induced podocyte injury.

Accession Number	Protein Name	Fold Change in Expression	Protein *M*_W_	Protein PI
P01027	Complement C3	1.278	186.4	6.7

**Table 3:** List of primers used in conventional PCR studies.

Gene	Primer Sequence (5′–3′)	Product (bp)
*C1qb*	Forward ACGGGGCTACACAGAAAGTC	123
NM_009777.2	Reverse TGCGTGGCTCATAGTTCTCG	
*C1s*	Forward TGGACAGTGGAGCAACTCCGGT	256
NM_144938.2	Reverse GGTGGGTACTCCACAGGCTGGAA	
*C1r*	Forward GCCATGCCCAGGTGCAAGATCAA	313
NM_023143.3	Reverse TGGCTGGCTGCCCTCTGATG	
*C2*	Forward CTCATCCGCGTTTACTCCAT	178
NM_013484.2	Reverse TGTTCTGTTCGATGCTCAGG	
*C3*	Forward AGCAGGTCATCAAGTCAGGC	167
NM_009778.2	Reverse GATGTAGCTGGTGTTGGGCT	
*C4*	Forward TCGCAGACATCACCCTCCTA	274
NM_009780.2	Reverse CTCTTGGTGGGTGCAGCATA	
*CFB*	Forward CTCCTCTGGAGGTGTGAGCG	264
NM_001142706.1	Reverse GGTCGTGGGCAGCGTATTG	
*CFD*	Forward TCAATCATGAACCGGACAAC	180
NM_013459.3	Reverse ATTGCCACAGACGCGAGAGC	
*CFP*	Forward GAGAGGCCCAGCAATCACAG	141
NM_008823.3	Reverse AGCGGCTTCGTGTCTCCTTA	
*MBL1*	Forward AGGGAGAACCAGGTCAAGGGCT	414
NM_010775.2	Reverse ACTGCCCTTCAGTCGCCTCGT	
*MBL2*	Forward CCCTGCCTGCAGTGACACCA	443
NM_010776.1	Reverse AGCACCCAGTTTCTCAGGGCT	
*Fcn1*	Forward AGGAGAAAAAGCTGAGCCGT	126
*NM_007995.3*	Reverse CCACTGCATTGCTCTGGGTA	
*Fcn*	Forward AGTGCCACACTTCCAACCTG	137
NM_010190.1	Reverse CTAGATGAGCCGCACCTTCA	
*MASP*	Forward GGCCTGAACCTGTATTCGG	497
NM_001003893.2	Reverse CTGGCCTGAACAAAGGGCT	
*C5*	Forward AGGGTACTTTGCCTGCTGAA	173
NM_010406.2	Reverse TGTGAAGGTGCTCTTGGATG	
*C6*	Forward GCGCTTCAAGAGTATGCAGC	285
NM_016704.2	Reverse CTTGCCACCACAGCTTTGTC	
*C7*	Forward AGAGGGCAGAGCATCTCCAT	196
NM_001243837.1	Reverse ATCCATTGCCCATTAGCTTC	
*C8b*	Forward TGTGACCAGAACCAAACGCT	229
BC096382.1	Reverse GTAGATGCCCCCAAGTACGG	
*C9*	Forward CACCTTAGCCCTTGCCATCT	354
NM_013485.1	Reverse TCTCCACAGTCGTTGTCACC	
*CFH*	Forward CGTGAATGTGGTGCAGATGGG	248
*NM_009888.3*	Reverse AGAATTTCCACACATCGTGGCT	
*CFI*	Forward TTCCACTGGGTGTTCGTGAC	126
NM_007686.2	Reverse TAAAGGCACACTCCGCCAAA	
*DAF*	Forward ACGGTACGTCATCCAACGAG	315
NM_010016.2	Reverse AGCCAACGAAGAGTTACGAAGA	
*CD46*	Forward CCAGGGCCAGATAAGTTTTC	153
NM_010778.3	Reverse TATTTCGCCAGCTCCTGATA	
*CD5*9	Forward TAAGTGAGTTCCTGGCAACC	152
NM_007652.5	Reverse AGGGCCTGTGAAGATTATGA	
*C4bp*	Forward GCCAGCAAGTGACGTGAATC	292
NM_007576.3	Reverse TTTGCCTCGGACCTCACAAG	
*Serping 1*	Forward TCTGCGACTGTCTGCTCAGT	216
NM_009776.3	Reverse AGCTCTCTCTGCTTTTCGCT	
*Crry*	Forward CCAAACAATGTGGGGATAGCAG	306
L19874.1	Reverse TGTCTGCCAAAGTGGGCTTA	
*CR2*	Forward CCTGCTCCTCTCTGTAAACT	162
NM_007758.2	Reverse GATCTGACTGCTTCCACTCA	
*C3aR*	Forward AGAGGTGACTCATGGAAAGGC	167
AF053757.1	Reverse ACTGATGATCTGCGAGCCAC	
*C5aR*	Forward TATCAGGTGACCGGGGTGAT	248
NM_007577.4	Reverse GTCGTGGACGGAGTGAAAGT	
*C1qR*	Forward AGCAAGCCGACACATGAAGA	114
NM_010740.3	Reverse CAGCACCAGCAAGAGTGAGA	
*Protein S*	Forward GTGAGGGTATCCCAGTGTGC	225
NM_011173.2	Reverse CATCACGAAGCGCAATCAGG	

## Supplementary Materials

Supplementary materials can be found at http://www.mdpi.com/1422-0067/17/4/471/s1.
